# Correlation between admission blood glucose, fibrinogen, and slow blood flow during primary PCI for acute ST segment elevation myocardial infarction

**DOI:** 10.3389/fcvm.2024.1478743

**Published:** 2024-12-03

**Authors:** Wu Zufei, Su Wentao, Shi Chen, Bai Da Xu, Gang Jun Zong, Gang-Yong Wu

**Affiliations:** ^1^ Department of Cardiology, The 904th Hospital of the PLA Joint Logistics Support Force, Wuxi, Jiangsu, China; ^2^ Department of Cardiology, Xuancheng People’s Hospital, Xuanchen, Anhui, China; ^3^ Department of Cardiology, Wuxi Clinical College of Anhui Medical University, Wuxi, Jiangsu, China; ^4^ Department of Cardiology, Wuxi No.5 People’s Hospital, Wuxi, Jiangsu, China

**Keywords:** admission blood glucose, fibrinogen, acute ST segment elevation myocardial infarction, percutaneous coronary intervention, slow blood flow

## Abstract

**Backgroud:**

Coronary slow flow (CSF) is a common phenomenon of coronary microcirculation dysfunction, and is closely related to elevated blood glucose and fibrinogen (FIB) levels. However, whether immediate blood glucose and FIB levels affect coronary blood flow during primary percutaneous coronary intervention (PCI) remains unclear.

**Objective:**

To explore the correlation between admission blood glucose (ABG), fibrinogen (FIB) and slow blood flow during primary PCI for acute ST segment elevation myocardial infarction (STEMI).

**Methods:**

A total of 497 patients who underwent coronary angiography in the cardiology department of the 904th Hospital of the Joint Logistics Support Force from December 2018 to December 2022 due to STEMI were selected consecutively, and then were divided into two groups based on whether slow blood flow occurred during primary PCI: slow blood flow group (*n* = 117) and control group (*n* = 380). Detecting the ABG, FIB and other indicators of patients in each group, and using logistic regression analysis and receiver operating characteristic (ROC) curve to analyze independent risk factors for slow blood flow during primary PCI, and further evaluating the prognosis of patients.

**Results:**

The levels of ABG and FIB in patients with slow blood flow were significantly higher than those in the control group (*P* < 0.05). The results of multivariate logistic regression analysis suggested that FIB and ABG were independent risk factors for slow blood flow during primary PCI (both *P* < 0.05). ROC curve analysis showed that ABG, FIB, and their combination all had predictive value for slow blood flow during primary PCI (all *P* < 0.05), and the area under the curve (AUC) of the combined indicator was higher than that of any single indicator, with statistical significance (*P* < 0.05). KM curve analysis suggested that the prognosis of patients in slow blood flow group were poor.

**Conclusion:**

Both elevated ABG and FIB could predict slow blood flow during primary PCI, and the diagnostic value of the combined indicator was superior to that of any single indicator, which could be used for the evaluation of slow blood flow during primary PCI, so as to evaluate the prognosis of patients with STEMI.

## Introduction

Coronary slow flow (CSF) is a phenomenon of coronary microcirculation dysfunction, mainly characterized by delayed distal vessel perfusion in the absence of significant epicardial coronary artery stenosis (1). The incidence of CSF in patients undergoing coronary angiography and intervention treatment is reported to be 1%–7% (2). The pathogenic factors mainly include microvascular (3) and endothelial dysfunction (4), small vessel rupture, chronic systemic or local inflammation (5), diffuse atherosclerosis (6), platelet dysfunction (7), impaired lipid metabolism (8), obesity, broken heart syndrome and so on (9, 10). Researches have shown that plasma fibrinogen (FIB) was a representative biomarker of chronic inflammation, playing a key role in the progression of inflammation, platelet activation, upregulation of adhesion molecule expression, angiogenesis, and macrophage infiltration (11, 12). Elevated plasma fibrinogen levels in an inflammatory state can promote platelet aggregation, affect coronary flow viscosity, lead to endothelial injury, and impair vascular constriction and dilation. Furthermore, plasma fibrinogen levels are also an independent predictor of CSF (13–15).

High blood glucose was identified as the primary cause of vascular complications in diabetes. Elevated blood glucose levels have been found to adversely affect the production of nitric oxide, leading to impaired endothelial-dependent vasodilation in both diabetic patients and experimental diabetic animals, ultimately resulting in CSF (16–18). A study conducted by Samad Ghaffari and colleagues discovered that fasting blood glucose was an independent risk factor for slow flow in patients undergoing coronary angiography (19). However, there are limited researches on the correlation between FIB, blood glucose, and CSF, and no reports on the correlation between admission blood glucose (ABG) and CSF. In theory, ABG has potential diagnostic value for CSF. Therefore, this study aims to explore the value of ABG, FIB, and their combined application in evaluating CSF by using the Thrombolysis in Myocardial Infarction (TIMI) flow grading during coronary angiography, and further investigate the correlation between ABG, FIB and CSF and adverse outcomes in patients with STEMI after primary PCI.

### Patients and methods

#### Patients

**Figure 1:**
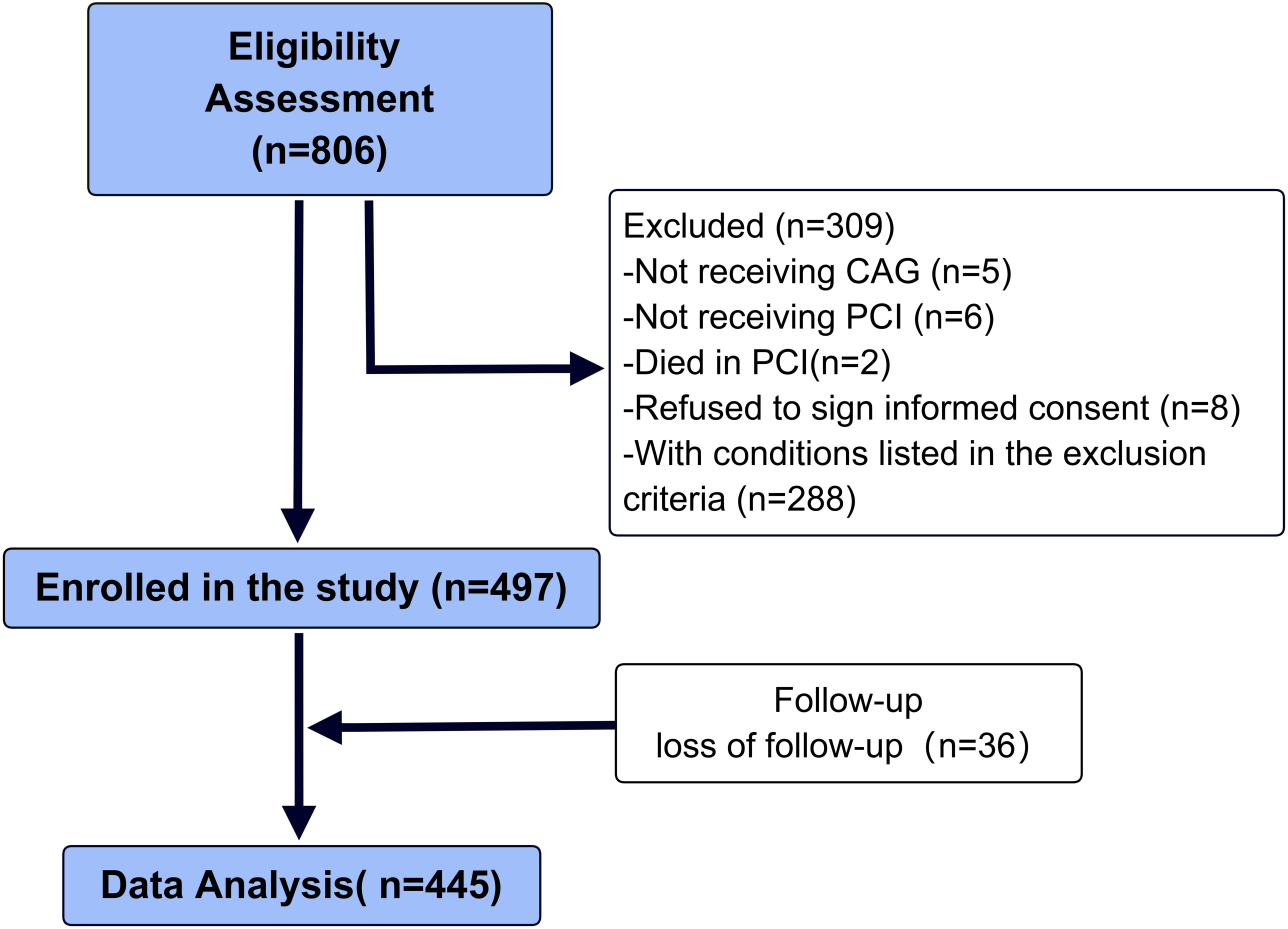
Flowchart of enrollment.

We conducted a retrospective analysis of a total of 806 patients admitted to the Cardiology Department of the 904th Hospital of the Joint Logistic Support Force due to chest pain from December 2018 to December 2022. The diagnosis of acute ST-segment elevation myocardial infarction (STEMI) was based on the 2017 ESC Guidelines (20). The main inclusion criteria were: (1) patients aged 30–85 years who were admitted due to acute chest pain and diagnosed with STEMI, and underwent primary coronary angiography and coronary artery stent implantation; (2) patients who were conscious, able to communicate naturally, and had no severe neurological or mental disorders. Upon admission, all subjects received standardized dual antiplatelet therapy (aspirin + ticagrelor/clopidogrel), and at discharge, they were given dual antiplatelet therapy (aspirin + ticagrelor or clopidogrel) and lipid-lowering therapy (atorvastatin or rosuvastatin) according to the guidelines. If there were no contraindications, ACEI/ARB and beta-blockers were also administered to inhibit myocardial remodeling. The main exclusion criteria were: (1) Patients with a history of myocardial infarction, coronary intervention, or coronary artery bypass graft surgery (*n* = 100); (2) Patients with accompanying pulmonary embolism, aortic dissection, acute or chronic nephritis, and other systemic diseases (*n* = 0); (3) Patients with blood system diseases, malignant tumors, autoimmune diseases, acute or chronic infectious diseases (*n* = 73); (4) Patients who underwent primary PCI for non-ST-segment elevation myocardial infarction (NSTEMI), variant angina, or other conditions (*n* = 110); (5) Patients with complications such as coronary artery dissection or perforation during PCI (*n* = 5). Ultimately, a total of 288 cases were excluded (Figure 1).

#### Grouping

According to whether slow flow occurred during primary PCI, the patients were divided into two groups: the slow flow group (*n* = 117) and the control group (*n* = 380) In order to study the occurrence of adverse events in different subgroups, discharged patients were further divided into the FIB-h (FIB ≥ 3.085 g/L) group and FIB-l (FIB < 3.085 g/L) group, the ABG-h (ABG ≥ 6.79 mmol/L) group and ABG-l (ABG < 6.79 mmol/L) group based on the results of the receiver operating characteristic (ROC) curve, respectively.

#### Research method

The patient's age, height, weight, as well as systolic blood pressure (SBP) and diastolic blood pressure (DBP) upon admission, were collected. Serum creatinine, blood glucose levels, and other parameters were measured using an automatic biochemical analyzer (Olympus, Japanese). Estimated glomerular filtration rate (eGFR) was calculated using the following formula: eGFR (ml/min/1.73m2) = (140-age) × weight/0.818 × creatinine concentration (umol/L), with the female value scaled by 0.85. Body mass index (BMI) was calculated as weight (kg) divided by the square of height (m). Plasma levels of cardiac troponin I (cTnI, Getein Biotechnology Co., Ltd.) and N-terminal pro-brain natriuretic peptide (NT-proBNP, Getein Biotechnology Co., Ltd.) were immediately measured after admission using a quantitative immunofluorescence assay.

Primary PCI was performed according to standard PCI methods. Based on the thrombus load, operators chose to use thrombus aspiration and platelet glycoprotein IIb/IIIa receptor antagonists. After balloon dilation, nitroglycerin was routinely injected into the coronary artery to obtain true lumen diameter. Stent diameter was selected based on the normal segment diameter of the culprit vessel (1–1.1:1). Immediate assessment of target vessel blood flow was performed after stent implantation by using TIMI flow grading. In this study, TIMI grades 0–2 were classified as the slow flow group, and TIMI grade 3 was classified as the normal flow group. For patients without contraindications, aspirin (300 mg, followed by 100 mg daily), ticagrelor (180 mg, followed by 90 mg twice daily), beta-blockers, statins, and renin-angiotensin system inhibitors (RASi) were administered. All patients were followed up in the clinic or by telephone after discharge. The primary outcome during the follow-up period was hospitalization due to heart failure, and the secondary outcome was the major adverse cardiovascular events (MACE) including cardiovascular death, hospitalization due to heart failure (HF), recurrent myocardial infarction, or unstable angina (UA), excluding readmission for selective PCI for patients with multivessel disease after discharge.

This clinical study has been approved by the Medical Ethics Committee of 904 Hospital of the Joint Logistics Support Force of the People's Liberation Army of China, with approval number 20160102. Each patient has signed a written consent form, stored their information in the hospital database, and used it for analysis and research.

### Statistic analysis

Continuous variables are expressed as mean and standard deviation or median and quartile, and group differences were compared using analysis of variance and least significant difference. Categorical variables are presented as frequency and percentage, and group differences were compared using the chi-square test. Logistic regression analysis was conducted to examine the relationship between FGB, FIB levels and the occurrence of slow flow during PCI and adverse events after discharge. The predictive value of FGB and FIB for slow flow was evaluated using ROC curves, and the cutoff point was selected based on the sum of sensitivity and specificity. The statistical significance level was set at *p* < 0.05. All statistical analyses were performed using SPSS software.

## Results

### Patient characteristics

In present study, 5 patients refused to undergo coronary angiography, 6 patients refused PCI, 2 patients died in PCI, and 8 patients refused to sign informed consent. Finally, 497 patients (366 males and 131 females) with an average age of 59 (59.8 ± 13.6) years were included in the study. During the discharge follow-up, 36 patients were lost to follow-up, 16 patients died resulting from complications during postoperative hospitalization, and 445 patients were followed up via phone and/or outpatient visits after discharge. All patients received dual antiplatelet therapy, statins, Renin-angiotensin system inhibitors (RASI), and beta-blocker treatment, with three patients not receiving RASI due to intolerance to blood pressure. The median follow-up time of 375 (295.5, 611) days.

### ABG, FIB, and slow blood flow

All enrolled patients were divided into two groups based on whether slow flow occurred during the operation, which were named as the slow flow group (*n* = 117) and the control group (*n* = 380). As shown in Table 1, the patients in the slow flow group were older, had diminished cardiac function, and a greater occurrence of diabetes. Additionally, neutrophil (NE), white blood cell (WBC), Troponin I (TnI), brain natriuretic peptide (BNP), serum creatinine (Scr), estimated glomerular filtration rate (eGFR), cystatin C (CysC), prothrombin time activity (PT%), international normalized ratio (INR) of prothrombin time, ABG, FIB, and the total number of lesioned vessels at admission were significantly higher in the slow flow group compared to the control group (all *P* < 0.05).

**Table 1:** Comparison of clinical and biochemical data of hospitalized patients with different group.

Variables	Control group (*n* = 380)	Slow-reflow group (*n* = 117)	*z/X^2^*	*P*
Male, *n* (%)	275 (72.37)	91 (77.78)	1.349	0.246
Age, (years)	59.5 (50,68)	65 (52,72.5)	−2.642	0.008
Heart rate	78 (68,88)	78 (68,92)	−0.717	0.473
SBP, (mmHg)	127 (117.25,140)	128 (120,146)	−0.358	0.72
DBP, (mmHg)	78.5 (70,86)	80 (70,89)	−1.225	0.22
BMI, (kg/m2)	24.221 (22.857,26.281)	24.802 (23.713,27.486)	−2.201	0.028
Killip class	0 (0,1)	1 (0,2)	−7.326	<0.001
Hypertension, *n* (%)	225 (59.21)	78 (66.67)	2.090	0.148
Diabetes, *n* (%)	85 (22.37)	44 (37.61)	10.809	0.001
Smoking, *n* (%)	270 (71.05)	79 (67.52)	0.533	0.465
Biochemical parameters				
HGB, (g/L)	140 (128,149)	141 (128.5,155)	−1.409	0.159
RBC, (× 109/L)	4.5 (4.1925,4.8975)	4.66 (4.16,5.025)	−1.462	0.144
NE, (× 109/L)	7.09 (5.17,9.4025)	7.57 (5.68,11.26)	−2.321	0.02
LY, (× 109/L)	1.54 (1.16,2.1375)	1.5 (1.01,1.905)	−1.388	0.165
MONO, (× 109/L)	0.645 (0.5,0.87)	0.66 (0.49,0.92)	−0.462	0.644
PLT, (× 109/L)	208 (165.25,244.75)	203 (162,240)	−0.381	0.703
WBC, (× 109/L)	9.755 (7.523,12.158)	10.57 (8.705,13.65)	−2.794	0.005
MCHC, (g/L)	342 (333,347)	341 (331.5,348.5)	−0.755	0.45
cTnI, (ng/ml)	0.396 (0.0453,3.913)	1.31 (0.135,7.75)	−2.471	0.013
NT-proBNP, (pg/ml)	376 (133.25,1109.8)	733.1 (309,1674)	−3.834	<0.001
Scr, (umol/L)	71.5 (62,83)	76 (64,90)	−2.19	0.029
eGFR, (ml/min/1.73 m^2^)	95.637 (78.364,120.629)	88.877 (63.922,113.854)	−2.251	0.024
CysC, (mg/L)	0.87 (0.76,1.028)	0.92 (0.805,1.135)	−1.97	0.049
CRP, (mg/L)	7.15 (2.645,19.875)	8.8 (3.65,27.205)	−1.841	0.066
DDimer, (mg/ml)	0.3 (0.19,0.5)	0.42 (0.24,0.86)	−4.023	<0.001
ABG, (mmol/L)	5.945 (5.193,7.265)	7.88 (6.095,10.880)	−6.567	<0.001
Prothrombin time activity	104.2 (89.8,118.4)	97.2 (85.2,115.05)	−2.037	0.042
INR	1.05 (0.983,1.128)	1.08 (1.02,1.15)	−2.458	0.014
APTT, (s)	29.7 (27.2,33.28)	29.7 (26.8,33.15)	−0.462	0.644
PT, (s)	12.05 (11.4,12.9)	12.4 (11.7,13.2)	−2.403	0.016
TT, (s)	17.65 (16.6,19.2)	17.8 (16.1,20)	−0.429	0.668
FIB, (g/L)	2.98 (2.435,3.58)	3.53 (2.875,4.6)	−5.126	<0.001
Procedure parameters				
Onset-to-balloon, (hours)	3 (2,6)	3 (2,6)	−1.01	0.312
Stent diameter, (mm)	3 (2.75,3.25)	3 (2.75,3.25)	−0.452	0.651
Stent length,(mm)	36 (28,51)	37 (29,56)	−1.5	0.134
Thrombus aspiration, *n* (%)	82 (21.58)	33 (28.21)	2.208	0.137
Post-dilation frequency, (*n*)	5 (3,6)	4 (3.5,6)	−0.181	0.856
Number of diseased coronary arteries, (*n*)	2(1,3)	3(2,3)	−3.127	0.002

BMI, body mass index; SBP, systolic blood pressure; DBP, diastolic blood pressure; HGB, hemoglobin; RBC, red blood cell; NE, neutrophil; LY, lymphocyte; MONO, monocyte, PLT platelet; WBC, white blood cell; MCHC, mean corpuscular hemoglobin concentration; cTnI, cardiac troponin I; NT-proBNP, N-terminal pro-brain natriuretic peptide; Scr, serum creatinine; eGFR, estimated glomerular fltration rate; CysC, cystatin C; CRP, C-reactive protein; ABG, admission blood glucose; INR, international normalized ratio; APTT, activated partial thromboplatin time; PT, prothrombin time; TT, thrombin time; FIB, fibrinogen.

**Figure 2:**
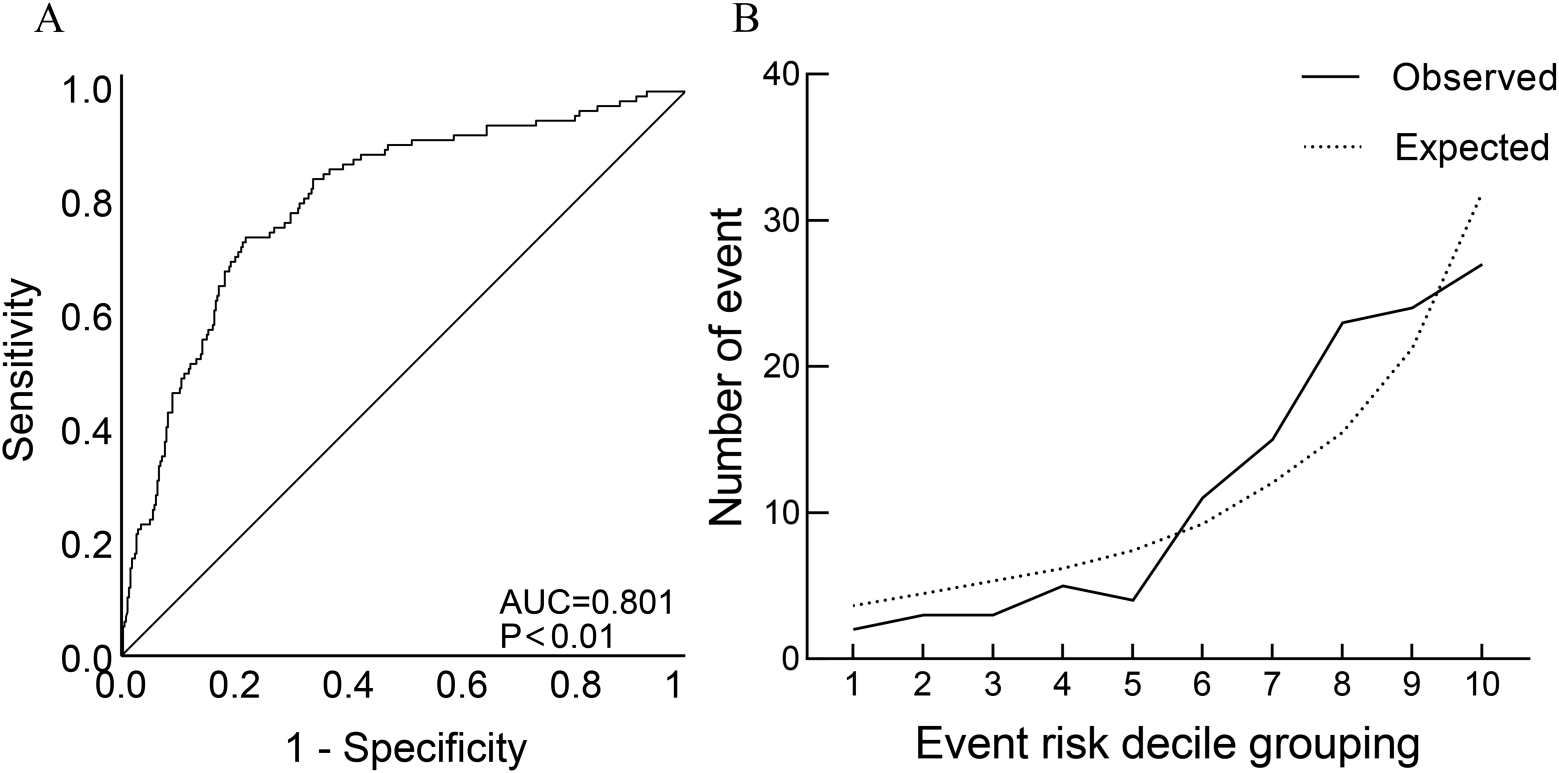
Discrimination ROC graph and calibration graph of multivariate logistic regression analysis of slow-refow. **(A)** Model discrimination ROC graph; **(B)** model calibration graph: sort the predicted probabilities of each research object from small to large and divide them into ten groups according to decile. The actual observation value and model predicted value of each group are expressed in the form of coorsdinate points, and the model calibration ability is judged as a whole. The closer the predicted curve is to the actual observation curve, the better the calibration capability of the model.

Based on the general data comparison, using *P* < 0.05 as the standard, significant parameters from the general data comparison (NE, WBC, TnI, BNP, Scr, eGFR, CysC, PT%, INR, ABG, FIB, lesion vessel number) were included in Univariate logistic analysis. The results showed that age, diabetes, Killip classification, NE, WBC, PT, ABG, FIB and lesion vessel number were risk factors for slow flow during primary PCI (all *P* < 0.05). Using *P* < 0.05 as the standard, significant parameters from Univariate logistic regression analysis were included in the collinearity test: there was no multicollinearity between the variables (tolerance was much greater than 0.1, variance inflation factor was less than 10). Age, diabetes, Killip classification, NE, WBC, PT, ABG, FIB, and number of diseased vessels were included in a multiple logistic analysis. The results indicated that Killip classification (*OR* = 1.442, 95%*CI* 1.194–1.741), FIB (*OR* = 1.488, 95%*CI* 1.211–1.731), ABG (*OR* = 1.970, 95%*CI* 1.115–1.285) were independent correlation factors for slow flow during primary PCI (all *P* < 0.05), as shown in Table 2. Model discrimination test: Predictive model ROC curve [AUC = 0.801, 95%*CI* 0.754–0.848, *P* < 0.01], AUC > 0.75 suggests good discriminatory ability of the predictive model. Model calibration test: The Hosmer-Lemeshow test was used to evaluate the calibration ability of the predictive model, and the results showed Hosmer-Lemeshow *Χ^2^* = 14.18, *P* = 0.077, demonstrating that the predictive model had good calibration ability, as depicted in Figure 2.

**Table 2:** Univariate and multivariate logistic regression analysis of factors affecting slow-refow phenomenon in primary PCI procedure.

	Univariate logistic				Multivariate logistic			
	B	Wald	OR(95%CI)	*P*	B	Wald	OR(95%CI)	*P*
Diabetes	−0.738	10.561	0.478 (0.306–0.746)	0.001				
Age	0.02	6.228	1.02 (1.004–1.036)	0.013				
BMI	0.066	4.194	1.068 (1.003–1.138)	0.041				
Killip class	0.552	40.314	1.736 (1.464–2.059)	0	0.366	14.497	1.442（1.194–1.741）	<0.001
NE	0.085	10.818	1.089 (1.035–1.145)	0.001				
WBC	0.084	11.009	1.088 (1.035–1.144)	0.001				
PT	0.144	5.567	1.155 (1.025–1.301)	0.018				
FIB	0.424	25.417	1.528 (1.296–1.801)	0	0.37	16.522	1.448（1.211–1.731）	<0.001
ABG	0.206	37.094	1.229 (1.150–1.314)	0	0.18	24.60	1.97（1.115–1.285）	<0.001
Number of diseased coronary arteries	0.278	9.667	1.32 (1.108–1.573)	0.002				

BMI body mass index, NE Neutrophil, WBC White blood cell, PT Prothrombin time, FIB Fibrinogen, ABG admission blood glucose.

**Figure 3:**
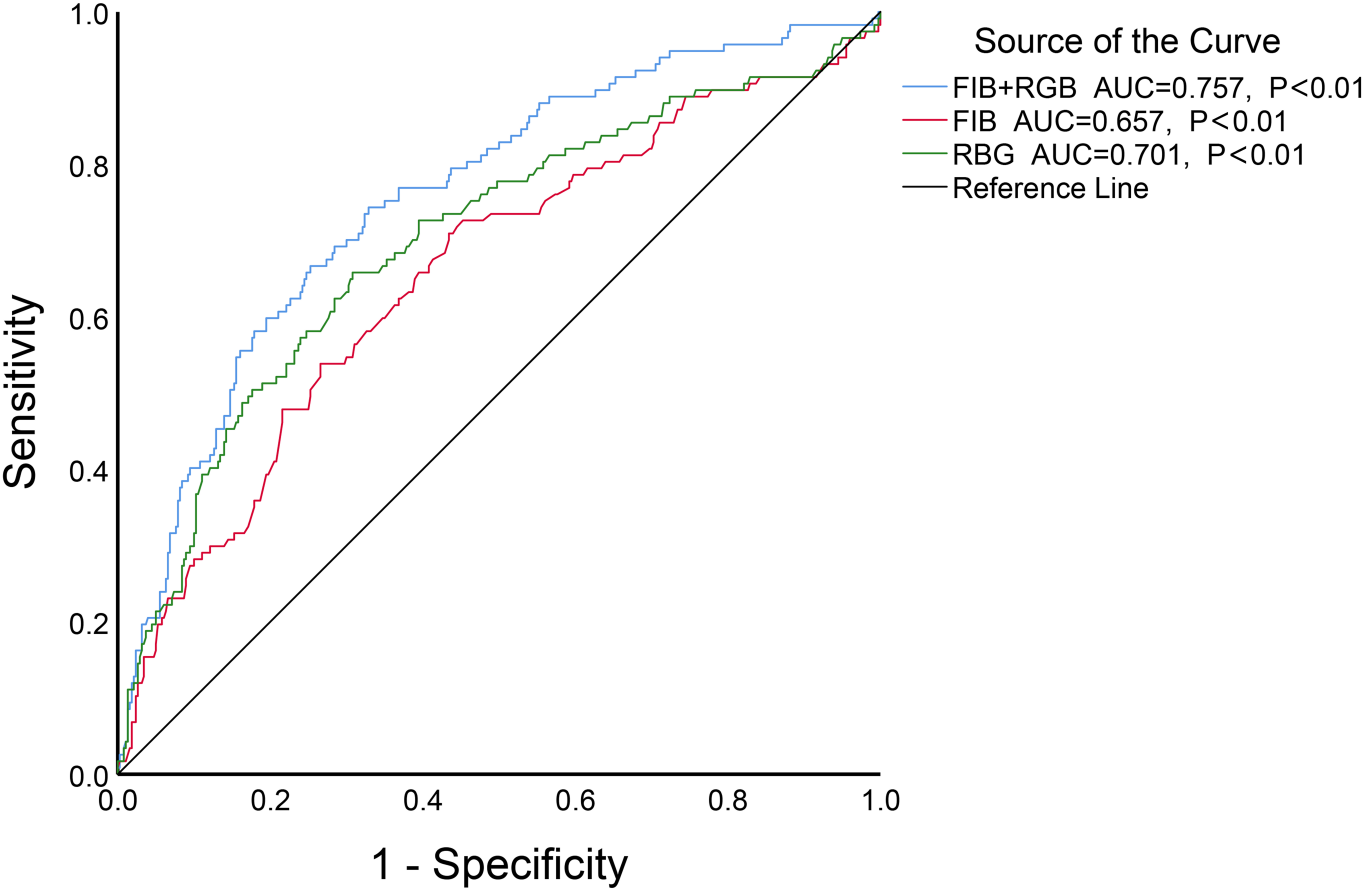
The ROC curve analysis of FIB, ABG and the combination of the two to predict CSF.

The results showed of ROC curve that when FIB was set at a threshold of 3.085 g/L, it had the highest predictive efficacy for CSF, with a sensitivity of 70.9%, specificity of 56.6%, AUC = 0.657, 95%*CI* 0.598–0.716, *P* < 0.01. When ABG was set at a threshold of 6.79 mmol/L, the sensitivity was 65.8%, specificity was 69.2%, and AUC = 0.701, 95%*CI* 0.643–0.758, *P* < 0.01. When both FIB and ABG were combined, they were better at predicting CSF (AUC = 0.757, 95%CI 0.706–0.808, *P* < 0.01), as depicted in Figure 3. Furthermore, the combined FIB and ABG had better predictive ability for CSF than either single indicator, and the differences were both statistically significant [RBG (Z = 1.97, *P* < 0.05), FIB (Z = 3.753, *P* < 0.05), Table 3].

**Table 3:** Comparison of the AUC of related indexes of coronary heart disease.

Variables	Difference between areas	SE	95% CI	Z	*P*
FIB + ABG VS ABG	0.0546	0.0277	0.000284–0.109	1.97	0.049
FIB + ABG VS FIB	0.0986	0.0263	0.0471–0.150	3.753	<0.001
FIB VS ABG	0.0440	0.0458	−0.0457–0.134	0.962	0.336

FIB, fibrinogen; ABG, admission blood glucose.

### ABG, FIB, slow blood flow and prognosis

After the patient's discharge, regular follow-up was conducted, with a total of 445 cases (92.5%) completing the follow-up, with a median follow-up time of 375(295.5, 611) days. During the follow-up, a total of 161 cases (36.18%) experienced MACE, including 11 cases of all-cause mortality, 10 cases of cardiac death, 123 cases of readmission due to recurrent ischemic chest pain (MI of UA), and 41 cases of hospitalization due to HF. According to the ROC curve results, the patients were divided into the FIB-h (FIB ≥ 3.085 g/L) group and the FIB-l (FIB < 3.085 g/L) group. Kaplan-Meier survival analysis showed there were no statistically significant differences in the risk of post-discharge MACE, readmission due to HF or recurrent ischemic chest pain between the different FIB groups after discharge (all *P* > 0.05). This suggests that FIB may not be significantly correlated with the long-term prognosis after primary PCI for STEMI. Similarly, all patients were divided into the ABG-h (ABG ≥ 6.79 mmol/L) group and the ABG-l (ABG < 6.79 mmol/L) group. Kaplan-Meier survival analysis showed that compared the control group, the risk of readmission due to HF in patients with slow blood flow after discharge were statistically significant difference. However, there were no statistically significant differences in the risk of MACE and recurrent ischemic chest pain between the different ABG groups after discharge (all *P* > 0.05). Patients were also grouped based on whether they experienced slow blood flow during PCI, and Kaplan-Meier survival analysis showed that compared the control group, the risk of MACE, heart failure, and recurrent ischemic chest pain in patients with slow blood flow after discharge were statistically significant difference (all *P* < 0.05), indicating that patients with STEMI who experienced slow blood flow during primary PCI could have an unfavorable medium to long-term outlook (Supplementary Figures, S1, S2, S3).

## Discussion

The main mechanism by which high blood glucose damages endothelial function is the increase in oxidative stress and inflammation, which can induce an increase in reactive oxygen species and serve a crucial function in oxidative stress. This is primarily through the following mechanisms: High blood glucose increases the formation of advanced glycation end products within the cells through the polyol pathway, leading to increased expression of advanced glycation end products and their receptor ligands, resulting in the activation of protein kinase C subtypes and excessive activation of the hexosamine pathway, causing oxidative stress and inflammatory reactions (21, 22). Elevated blood glucose involves not only the hyperglycemic state but also metabolic disorders, including obesity, abnormal blood lipids, insulin resistance, and hypertension. High blood glucose can stimulate adipocytes to produce inflammatory cytokines such as tumor necrosis factor-α and interleukin-6, while reducing the production of the anti-inflammatory cytokine adiponectin, leading to increased inflammation and endothelial dysfunction, promoting the occurrence of slow flow (23). As far as we know, there is no reports regarding the association between admission blood glucose and CSF, and limited research on the correlation between fibrinogen and CSF. In the present study, we discovered that admission blood glucose in patients with STEMI was independently associated with the occurrence of slow flow. The results of the ROC curve suggested that admission blood glucose had a certain predictive value for CSF in patients with STEMI. However, it did not demonstrate significant predictive value for adverse events during the follow-up period. This may be due to the marked rise in blood glucose levels during acute myocardial infarction, which leads to further aggravation of oxidative stress and increased inflammatory response, thus inducing the occurrence of slow blood flow. In the follow-up phase, admission blood glucose could not present a state of inflammation or oxidative stress *in vivo*, thus providing no prognostic value.

Fibrinogen is an inflammatory marker that is associated with the pathophysiology, presence, severity, and prognosis of coronary artery disease. A study by Osman Kayapinar and colleagues found that fibrinogen is significantly associated with CSF (13). Fibrinogen can stimulate endothelial cell degeneration and disintegration, as well as increase the release of endothelial cell-derived growth factors (24). This indicates that fibrinogen plays a role in the stimulation of vascular inflammation, leading to endothelial dysfunction. Our study found that fibrinogen was an independent risk factor for slow flow during primary PCI for acute myocardial infarction, and the ROC curve analysis suggested that fibrinogen had a certain diagnostic value for slow flow.

Fibrinogen is closely related to glucose metabolism disorder which has been confirmed by numerous studies. Zhang et al. found that in ACS and DM patients, FIB is related to FBG and HbA1c. Similarly, in a study of 5,237 newly diagnosed patients with coronary heart disease, Liu and his colleagues found that FIB was significantly associated with both FBG and HbA1c with or without diabetes (25). High blood glucose leads to oxidative stress, inflammatory response, coagulation activation, and accelerated atherosclerosis mechanisms (16). In patients with prediabetes and type 2 diabetes, FIB synthesis is significantly promoted (26).

The Killip classification is commonly used to assess cardiac function in patients with acute MI, often indicating a decline in cardiac function. Research has shown that heart failure can induce microvascular inflammation, leading to the migration of monocytes and the release of transforming growth factor (TGF) *β*. This pro-inflammatory and pro-oxidative state may make the dysfunctional coronary microvascular system more susceptible to myocardial ischemia and microinfarction, as well as cause systemic multi-organ inflammation and microvascular dysfunction (27–29). Our study results indicate a significant correlation between higher Killip classification and coronary slow flow, consistent with previous research (29). The findings suggest that patients with elevated Killip classification are more likely to experience slow flow, which holds important value for interventional cardiologists in the preoperative assessment of coronary slow flow.

All these results indicate a significant inherent connection between FIB and high blood glucose. In our study, we combined FIB and ABG to predict the occurrence of slow blood flow in primary PCI for acute myocardial infarction, which may be more sensitive and accurate compared to individual indicators. Single and multiple logistic regression analysis results showed that FIB and ABG are not only risk factors for CSF but also independent risk factors for CSF, indicating the important role of FIB and ABG in the occurrence of CSF. ROC curve analysis suggested that the combined use of FIB and ABG has a higher predictive value for CSF compared to single indicators, with statistically significant differences. This also indicates that the combined use of FIB and AGB has greater guiding significance from the perspective of predicting the mechanism of CSF formation, and can improve the clinical diagnostic value. Admission blood glucose has a more flexible detection time window compared to fasting blood glucose, and results can be obtained immediately without long waiting times. It is significantly related to CSF and can replace fasting blood glucose in predicting CSF. Patients with diabetes are more likely to have slow blood flow, and our study showed that slow blood flow was associated with ABG. Therefore, for diabetic patients, whether it is only those with elevated ABG that are prone to slow blood flow needs to be further studied.

### Coronary slow flow and treatment

It is important to note that CSF does not necessarily equate to a low perfusion state. Slow flow refers to a decrease in coronary blood flow velocity and volume, typically reflected by a lower TIMI flow grade, but this does not mean that the coronary microcirculation is in a complete low perfusion state. Slow flow may be caused by microvascular dysfunction or localized vascular constriction, and although the flow velocity is slower, a certain level of blood perfusion may still be maintained, without necessarily resulting in a global low perfusion phenomenon (30, 31). However, in the current study, the prognosis of patients in the slow flow group was significantly worse compared to the control group. This means that for patients with acute myocardial infarction undergoing primary PCI, it is recommended to take appropriate measures during PCI to prevent slow flow.

The treatment of coronary slow flow remains challenging, but several potential therapeutic strategies have been proposed. Two recent studies, including the RAIN-Flow study, found that saline-induced coronary hyperemia may play a role in treating the slow flow/no-reflow phenomenon (32, 33). The results of this study demonstrated that saline-induced coronary hyperemia could promote microvascular perfusion, improve local blood flow, and reduce oxidative stress and inflammation, potentially alleviating slow flow. Another study showed that new oral hypoglycemic agents, such as SGLT2 inhibitors, reduce the risk of adverse events in patients with acute myocardial infarction (34, 35). In our study, ABG was closely associated with the occurrence of CSF. During long-term follow-up, the incidence of adverse events in patients with slow flow was significantly higher than those in the control group. Therefore, we hypothesize that SGLT2 inhibitors may improve the prognosis of patients with STEMI by reducing the occurrence of slow flow during primary PCI, which warrants further investigation.

### Study limitations

The limitations of this study include: (1) the primary use of TIMI blood flow as the criterion for defining CSF, which is somewhat subjective, and a more precise method was not used to define CSF, thus having certain limitations; (2) ABG and FIB were single preoperative blood sample test results, and dynamic testing was not carried out; (3) the number of case samples is relatively small, and further large-sample, multicenter studies are needed to explore the relationship between ABG, FIB, and CSF.

## Conclusion

In conclusion, AGB and FIB reflect the level of inflammation and oxidative stress in patients, and might predict the occurrence of slow blood flow in primary PCI for acute myocardial infarction. The combined application of both has higher diagnostic value, and has certain guiding significance for the clinical diagnosis and treatment of coronary slow blood flow.

## Data Availability

The raw data used in this research are not publicly available due to privacy protection and ethical approval considerations. However, the data can be made available upon reasonable request to qualified researchers, provided that all privacy and ethical guidelines are followed. Requests to access the datasets should be directed to Wu Zufei, wuzufei@126.com.

## Supplementary material

Supplementary Figure S1 Kaplan-Meier curve of ABG in patients with acute ST-segment elevation myocardial infarction. (A) Major Adverse Cardiovascular Events (B) Recurrent MI or UA (C) Heart failure.

Supplementary Figure S2 Kaplan-Meier curve of Fib in patients with acute ST-segment elevation myocardial infarction. (A) Major Adverse Cardiovascular Events (B) Recurrent MI or UA (C) Heart failure.

Supplementary Figure S3 Kaplan-Meier curve of slow blood flow in patients with acute ST-segment elevation myocardial infarction. (A) Major Adverse Cardiovascular Events (B) Recurrent MI or UA (C) Heart failure.
